# Towards making immunization programmes in Africa more successful: a primer on implementation research

**DOI:** 10.11604/pamj.supp.2025.51.1.46919

**Published:** 2025-06-13

**Authors:** Abdu Abdullahi Adamu, Chukwudi Arnest Nnaji, Echezona Edozie Ezeanolue, Charles Shey Wiysonge

**Affiliations:** 1 Vaccine-Preventable Diseases Programme, World Health Organization Regional Office for Africa, Djoue, BP 06, Brazzaville, Congo,; 2 School of Public Health and Family Medicine, University of Cape Town, Cape Town, South Africa,; 3 Malaria Consortium, London, United Kingdom,; 4 Center for Translation and Implementation Research, University of Nigeria, Nsukka, Nigeria,; 5 IVAN Research Institute, Nigeria

**Keywords:** Immunization, implementation research, Africa

## Abstract

There is a protracted lag in efforts to widen access and uptake of routine vaccination services in Africa. As a result, vaccine-preventable diseases continue to remain a major public health challenge. The increasing adoption of implementation research in global health is an opportunity for immunization programmes to leverage new tools in tackling long-standing implementation barriers to vaccination efforts. Implementation research in immunization programmes can strengthen how contextual barriers hindering vaccination efforts are identified and addressed with sustainable, context-appropriate, and practical strategies. In this primer, which is targeted at country-level immunization decision-makers and implementers, we discussed the meaning of implementation research and the value addition it can bring in fast-tracking programmatic success when integrated into all aspects of the immunization landscape.“

## Commentary

Vaccine-preventable diseases (VPD) are still among the topmost public health challenges in the World Health Organization (WHO) African Region [1]. According to the Global Burden of Disease Study 2019, VPDs account for a substantial proportion of pathogen-associated disability-adjusted life years (DALY) in the region [1]. DALY is a measure of the burden of disease which combines morbidity and mortality into a single metric [1]. Table 1 shows the ranking of the top three pathogens that contribute the most to pathogen-associated DALY in the WHO African Region [1]. The commonest infectious causes of illnesses and deaths include malaria, tuberculosis, measles, rotavirus, and Streptococcus pneumoniae, all of which are preventable with existing vaccines [1].

**Table 1:** ranking of pathogens based on Disability Adjusted Life Years (DALY) in each of the 47 countries in the WHO African Region

	Ranking based on DALY Burden		
Country	Rank 1	Rank 2	Rank 3
Algeria	S aureus	K pneumoniae	S pneumoniae
Angola	HIV/AIDS	Malaria	Tuberculosis
Benin	Malaria	Measles	S pneumoniae
Botswana	HIV/AIDS	Tuberculosis	S pneumoniae
Burkina Faso	Malaria	Tuberculosis	S pneumoniae
Burundi	Malaria	Tuberculosis	HIV/AIDS
Cabo Verde	HIV/AIDS	Tuberculosis	S pneumoniae
Cameroon	Malaria	HIV/AIDS	Tuberculosis
Central African Republic	Tuberculosis	Malaria	HIV/AIDS
Chad	Rotavirus	S pneumoniae	Malaria
Comoros	Tuberculosis	Malaria	S pneumoniae
Congo	HIV/AIDS	Malaria	Tuberculosis
Cote d'Ivoire	Malaria	HIV/AIDS	Tuberculosis
Democratic Republic of the Congo	Malaria	Tuberculosis	S pneumoniae
Equatorial Guinea	HIV/AIDS	Malaria	Syphilis
Eritrea	Tuberculosis	HIV/AIDS	S pneumoniae
Eswatini	HIV/AIDS	Tuberculosis	S pneumoniae
Ethiopia	Tuberculosis	HIV/AIDS	Malaria
Gabon	HIV/AIDS	Malaria	Tuberculosis
Gambia	HIV/AIDS	Tuberculosis	Malaria
Ghana	Malaria	HIV/AIDS	Tuberculosis
Guinea	Malaria	S pneumoniae	Tuberculosis
Guinea-Bissau	Measles	HIV/AIDS	Malaria
Kenya	HIV/AIDS	Tuberculosis	Malaria
Lesotho	HIV/AIDS	Tuberculosis	S pneumoniae
Liberia	Malaria	HIV/AIDS	Tuberculosis
Madagascar	Tuberculosis	Malaria	Rotavirus
Malawi	HIV/AIDS	Malaria	Tuberculosis
Mali	Malaria	S pneumoniae	K pneumoniae
Mauritania	Malaria	S pneumoniae	K pneumoniae
Mauritius	S aureus	HIV/AIDS	Schistosomiasis
Mozambique	HIV/AIDS	Malaria	Tuberculosis
Namibia	HIV/AIDS	Tuberculosis	S pneumoniae
Niger	Malaria	Measles	Rotavirus
Nigeria	Malaria	S pneumoniae	HIV/AIDS
Rwanda	Malaria	Tuberculosis	HIV/AIDS
Sao Tome and Principe	Malaria	S aureus	S pneumoniae
Senegal	Malaria	Tuberculosis	K pneumoniae
Seychelles	S aureus	S pneumoniae	Escherichia coli
Sierra Leone	Malaria	HIV/AIDS	Tuberculosis
South Africa	HIV/AIDS	Tuberculosis	S aureus
South Sudan	Malaria	S pneumoniae	Tuberculosis
Togo	Malaria	HIV/AIDS	Rotavirus
Uganda	Malaria	HIV/AIDS	Tuberculosis
United Republic of Tanzania	HIV/AIDS	Malaria	Tuberculosis
Zambia	HIV/AIDS	Tuberculosis	Malaria
Zimbabwe	HIV/AIDS	Tuberculosis	Malaria

S aureus = Staphylococcus aureus; S Pneumoniae = Streptococcus pneumoniae; K pneumoniae = Klebsiella pneumoniae. The pink colour indicates pathogens for which vaccines are available as of 2024. This table was generated based on data obtained from the Global Health Data Exchange of the Institute for Health Metrics and Evaluation at University of Washington, Seatle, USA. It is based on the Global Burden of Disease Study 2019 and the full report for all 85 pathogens has been published [1]

Weak immunisation programmes are key contributors to the persistently high VPD burden in the African region. Although the success of national immunization programmes is measured across multiple indices, the ability to attain and maintain specific coverage benchmarks for new and existing vaccines at districts and national levels is one of the most important indicators. Evidence from the 2023 WHO - United Nations Children´s Fund (UNICEF) Estimates of National Immunization Coverage (WUENIC) showed that many individuals across multiple countries in the WHO African Region are not receiving nationally recommended vaccines [2]. For example, the cumulative DTP3 coverage for the African region is 74% [2]. Only 16 out of the 47 countries in the region reported DTP3 coverage of at least 90% [2]. Furthermore, the coverage for the first and second doses of measles-containing vaccines for the region in 2023 was 70% and 49%, respectively [2]. Since many African countries have large birth cohorts, the disruption caused by the COVID-19 pandemic resulted in further accumulation of unvaccinated (i.e. zero-dose) and under-vaccinated children [2].

### Why does implementation context matter in immunization programmes?

The observed gaps in vaccination coverage signal an underlying problem concerning how vaccination efforts are conducted across diverse communities. Implementing complex health system innovations like routine vaccination in complex health systems is not straightforward because of constantly interacting elements such as individuals, communities, institutions, and policies, among others [3]. The availability of essential building blocks like financial resources, human resources for health, highly efficacious vaccines and vaccination-related technologies, and policies do not necessarily guarantee the implementation success of vaccination efforts across diverse settings because the resultant behavior of interacting elements can be unpredictable, often varying from place to place [3]. The implementation success of routine vaccination can be vulnerable to the adverse influence of the dynamics of contextual determinants as well as the implementation climate in local health institutions responsible for providing vaccination services [4]. These determinants can undermine vaccination efforts in communities even when a country may have a well-resourced and highly experienced national immunization programme. For newer vaccines that are not yet in routine use and vaccines or immunization-related innovations at advanced stages of the development pathway, contextual determinants can influence decision-making regarding their integration into policies, thereby affecting timely use in regular practice settings when they become available. Therefore, without a thorough understanding of contextual determinants and how they interact, it will be difficult to know why vaccination efforts fail or succeed in specific settings. As implementation research (a field of study that promotes critical inquiry on contextual determinants and how to control them) gains more prominence in global health, it is critical to quickly integrate it into all aspects of the immunization landscape.

### What value can implementation research bring to the immunization landscape?

Implementation research is the scientific study of methods to promote the systematic uptake of evidence-based innovations like vaccines into routine healthcare settings with the goal of improving the quality and effectiveness of health services and care [5]. Implementation research is not concerned with generating evidence about the efficacy or effectiveness of vaccines or immunization-related innovations. In the immunization programme setting, implementation research offers new tools that can be used to enhance vaccination efforts. Several theories, models and frameworks can be used to understand and address contextual determinants that affect the successful implementation of vaccines [4]. For example, determinants frameworks such as Consolidated Framework for Implementation Research (CFIR) and Theoretical Domains Framework (TDF), among others, can aid context assessment [4], while evaluation models such as Reach Effectiveness Adoption Implementation and Maintenance (RE-AIM) can support the evaluation of practical implementation strategies [4]. Using these tools appropriately in the different aspects of the immunization landscape can make vaccines more impactful by enhancing the implementation success of vaccination efforts.

Implementation success (or failure) of vaccination efforts is conceptually distinct from optimal (or suboptimal) programme performance [6]. Vaccination efforts must first be implemented well across diverse communities for countries to attain their desired performance targets. Drawing on the framework for implementation outcomes by Proctor and colleagues, it can be deduced that the implementation success (or failure) of vaccination efforts is intermediate to the programme indicators [6]. Similarly, programme performance indicators are also proximal to population health indicators like DALYs. Visualizing the pathway from implementation to impact in this manner can form the basis of the theory of change of national immunization programmes, as it clearly explicates the importance of implementation.

mplementation research can be important in supporting immunization decision-makers and programme implementers to tackle implementation problems in at least three major ways. First, by facilitating a more holistic evaluation of contextual barriers that impede the successful implementation of vaccination efforts in specific settings. Second, by fostering systematic and collaborative solutions using tailored multicomponent strategies to address key contextual barriers that influence the success of vaccination efforts. When combined, these two can pave the way for agile learning in immunization programmes. Third, decision-making and policy actions regarding newer vaccines and other immunisation-related products and recommendations from global, regional, and national advisory groups can be bolstered with implementation evidence, thereby fast-tracking operationalization.

The success of implementation research relies on the extent of collaboration between decision-makers and researchers, as this enables policy-relevant inquiries that are aligned with real-world exigencies of immunization programmes [4]. As immunization practitioners, it is crucial to know how to clearly distinguish implementation research from other types of health service research to avoid resource wastage. For implementation research, the overarching question often focuses on how best to strengthen vaccination efforts in specific settings, with specific investigations into implementation context, strategies, and outcomes [4].

### How can implementation research be used to enhance immunization programmes?

Below are some illustrative examples of how implementation research can be applied for problem-solving in routine immunization contexts:

**Scenario 1**

***Implementation problem***

As an immunization programme manager, you regularly conduct monthly routine immunization coordination meetings with sub-national routine immunization officers to review progress and performance across districts. The health minister has expressed serious concerns about the national DTP3 coverage level, which was shown to be 54% in the recently released demographic and health survey. Since initiating the monthly meeting eight months ago, you noticed that 84 districts across five states have been reporting DTP1 coverage of less than 50%, with a DTP1 - DTP3 dropout rate of more than 10%. Feedback from the Routine Immunization officers backed by data from the logistics management system suggested that vaccines are available in the facilities in those districts. How do you approach this problem using implementation research?

***Solution approach***

Implementation research in practice settings is team-based and requires the participation of all relevant stakeholders. However, it is important to have an implementation scientist lead the process to ensure rigor and expert use of implementation research tools. If the immunization programme does not have an implementation scientist, collaboration can be established with local research institutions where such capacity exists. The problem described above can be addressed systematically through the identification of the context barriers in focus districts, co-creating practical strategies to address identified barriers, testing of the strategies in real-world practice settings, taking the strategies to scale in other districts, and integrating the strategies within the broader immunization policy. The methodological approach for each step can vary and depends on available resources.

**Step 1:** one way to systematically identify the contextual barriers is to use a rapid ethnographic assessment process guided by any relevant determinants framework. This can generate quick and actionable information about the implementation process and implementation determinants of routine vaccination in a sample of these districts.

**Step 2:** Stakeholders can co-create strategies to address identified barriers through intervention mapping during formative meetings. The steps involved in intervention mapping include conducting a needs assessment, creating matrices of change objectives, selecting theory-based intervention methods, integrating the strategies into a program, planning for adoption, implementation, and sustainability, and generating an evaluation plan [7]. Systems thinking can be used to map the dynamics of identified barriers, supporting proper strategy targeting. The Expert Recommendations for Implementing Change (ERIC) compilation can be consulted for suggested strategies and to align terminologies [8].

**Step 3:** process evaluation of the strategies should track implementation outcomes as well as key programmatic indicators. It is important to sustain stakeholder engagement throughout this period. Once there is clear real-world evidence that these implementation strategies are useful, then, they can be taken to scale in other districts with adaptation where necessary. Any adaptation should be properly documented.

**Scenario 2**

***Implementation problem***

As an immunization programme manager, you were approached by the National Immunization Technical Advisory Group to consider adding a booster dose of DTP containing vaccine to the national immunization schedule. The country has been experiencing bouts of diphtheria outbreaks and the epidemiological profile indicates that older children are most commonly affected. Moreover, this advice is in line with current WHO recommendations. Although the country recently began second year of life vaccination using measles containing vaccine, but the platform is still weak as MCV2 coverage is only 11%. How do you approach this problem using implementation research?

***Solution approach***

The decision to add a new vaccine to the national schedule is not easy as there are cost considerations, vaccine availability and human resources for health capacity, among many other issues that need to be thoroughly evaluated. Pilot implementation is appropriate as this vaccine will be a new addition to the national schedule, and it will target an age group that is not typically reached for routine vaccination in the country. Pilot implementation needs to be well-designed with clearly defined implementation outcomes. Implementation outcomes such as acceptability, adoption and feasibility need to be well understood to guide future planning for wider use. Quasi-experimental or experimental designs can be used for pilot implementation. Depending on resource availability, a hybrid effectiveness-implementation trial can be conducted [9]. This dual-purpose trial combines elements of clinical effectiveness research with implementation research [9]. There are three types of hybrid designs, but type 2 or type 3 are better suited for this scenario so that the researchers can focus on implementation either as a co-primary or primary aim [9].

### How can implementation research be integrated into immunization programmes?

**Figure 1:**
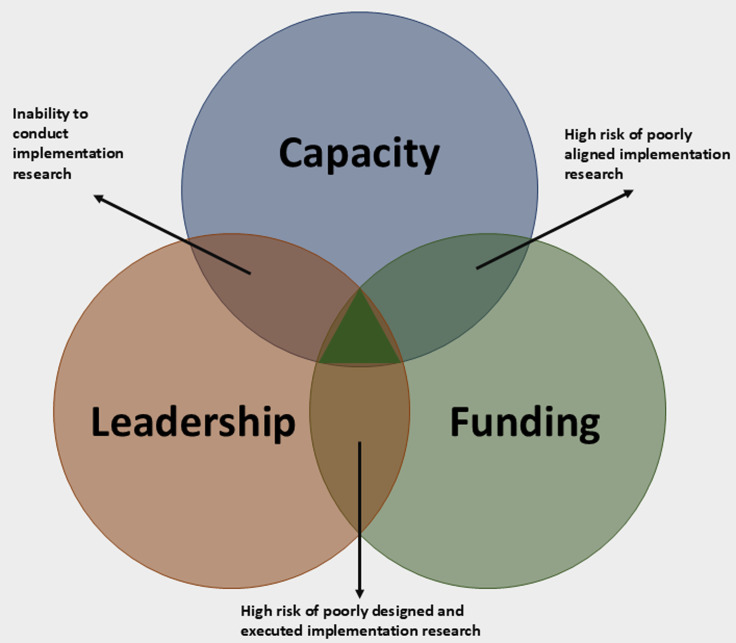
triad of top enablers for successful use of implementation research in immunization programmes

Leadership, capacity and funding are key enablers that are pivotal for the successful integration of implementation research in immunization programmes [10]. Leadership means immunization programme managers at national and sub-national levels exhibit a high degree of stewardship and ownership of implementation research. Capacity refers to having the requisite human resources with skills in designing and conducting implementation research within real-world immunization programme settings. Funding is the availability of finances either from external or internal sources, or both, that is dedicated to implementation research in immunization programmes. All three success levers must align to catalyze meaningful progress in integrating implementation research within immunization programmes. As shown in Figure 1, if there is capacity and funding but no leadership, immunization programmes could face the risk of conducting implementation research that is not aligned with priority policy issues. Also, if there is capacity and leadership but no funding, it will be difficult to execute an implementation study. Likewise, if there is leadership and funding but no capacity, there is a risk of conducting poorly designed implementation research.

## Conclusion

There are obvious advantages of implementation research that can help immunization programmes systematically tackle suboptimal coverage, inequity, and sustainability issues so that individuals in traditionally underserved areas have better access to and uptake of routine vaccination. Given that implementation research is still a relatively new field in the immunization ecosystem, there is a need for concerted efforts of governments, multilateral organizations and global health initiatives focused on vaccines and immunization to invest in capacity development so as to create a critical mass of implementation research experts that cuts across decision-makers and programme implementers within immunization programmes in the WHO African Region. Also, national immunization policies must explicitly encourage the use of implementation research in immunization programmes.
